# Expression and regulation of recently discovered hyaluronidases, HYBID and TMEM2, in chondrocytes from knee osteoarthritic cartilage

**DOI:** 10.1038/s41598-022-22230-z

**Published:** 2022-10-14

**Authors:** Jun Shiozawa, Susana de Vega, Chiho Yoshinaga, Xang Ji, Yoshifumi Negishi, Masahiro Momoeda, Tomomi Nakamura, Hiroyuki Yoshida, Haruka Kaneko, Muneaki Ishijima, Yasunori Okada

**Affiliations:** 1 Department of Pathophysiology for Locomotive Diseases, Graduate School of Medicine, Juntendo University, 2-1-1 Hongo, Bunkyo-Ku, Tokyo, 113-8241 Japan; 2 Department of Medicine for Orthopaedics and Motor Organ, Graduate School of Medicine, Juntendo University, Tokyo, Japan; 3 Biological Science Research, Kao Corporation, Kanagawa, Japan; 4 Sportology Center, Juntendo University, Tokyo, Japan

**Keywords:** Diseases, Rheumatology

## Abstract

Destruction of articular cartilage in osteoarthritis (OA) is initiated by depletion of the hyaluronan (HA)-aggrecan network, followed by degradation of the collagen fibrils. Previously, we reported the implications of HA-binding protein involved in HA depolymerization (HYBID), alias cell migration-inducing protein (CEMIP) and KIAA1199, for HA degradation. However, transmembrane protein 2 (TMEM2), which is ~ 50% homologous to HYBID, was discovered as another hyaluronidase, but their expression and regulation by OA chondrocytes remain elusive. Here we report that the absolute mRNA copy numbers of HYBID are significantly (7.1-fold) higher in OA cartilage than normal cartilage, whereas TMEM2 levels are not different between the groups. HA-degrading activity of cultured OA chondrocytes disappeared by siRNA-mediated knockdown of HYBID, but not TMEM2. HYBID expression was significantly up-regulated by treatment with interleukin-6 (IL-6) or tumor necrosis factor-α (TNF-α) and additively increased by the combined treatment. No significant changes in the TMEM2 expression were seen by the factors examined. IL-1α remarkably enhanced IL-6 production and increased HYBID expression when soluble IL-6 receptor was supplemented. These results demonstrate that in stark contrast to the constitutive expression of TMEM2 and its negligible HA-degrading activity, HYBID is overexpressed in OA cartilage and up-regulated by IL-6 and TNF-α in OA chondrocytes.

## Introduction

Osteoarthritis (OA), the most common joint disease in the elderly, is characterized by progressive degradation of articular cartilage extracellular matrix, which comprises mainly of the hyaluronan (HA)-aggrecan network and the collagen fibrils. The initial pathological change of the articular cartilage in OA joints is depletion of the HA-aggrecan network, which is followed by degradation of the collagen fibrils^1,2^. The matrix metalloproteinase (MMP) family members with collagenolytic activity such as MMP-1, MMP-13 and MMP-14 are responsible for degradation of fibrillar collagens, and a disintegrin and metalloproteinase with thrombospondin motifs 4 (ADAMTS4) and ADAMTS5 (aggrecanase-1 and aggrecanase-2, respectively) appear to play a central role in aggrecan degradation^1,3^.

Concerning HA degradation, hyaluronidases (HYALs), i.e., HYAL1, HYAL2, and PH20/SPAM1, were thought to be key enzymes^4^, and among them, HYAL-1 and HYAL-2 together with cell surface HA receptor CD44 were believed to play a key role in HA degradation^4^. However, since knockdown of these genes in human skin fibroblasts showed no changes in HA-degrading activity^5^, we sought molecules related to HA degradation by microarray analysis, and discovered that KIAA1199, which was reported as a deafness gene of unknown function^6^, is a novel molecule responsible for HA degradation^5^. KIAA1199 contributed to depolymerization of high-molecular-weight HA (HMW-HA) of 1,000–10,000 kDa into intermediate-sized HA fragments of 100–10 kDa in clathrin-coated vesicles independently from HYAL1 and HYAL2/CD44. We named this molecule as hyaluronan-binding protein involved in hyaluronan depolymerization (HYBID)^5,7^, but it is also known as cell migration-inducing protein (CEMIP)^8^. HYBID is a secreted protein composed of one G8, two GG and four PbH1 domains and selectively binds to HA^5^. Subsequently, Yamamoto et al.^9^ reported that transmembrane protein 2 (TMEM2), which is a murine type II transmembrane protein consisting of a transmembrane domain, one G8 domain, one GG domain and three PbH1 domains, acts as cell-surface hyaluronidase. They also showed that TMEM2 is more abundantly expressed than HYBID in various mouse tissues^9^ and in human tumor cell lines compared to human skin and lung fibroblasts^10^.

Our previous studies demonstrated that histamine and transforming growth factor-β1 (TGF-β1) up-regulates and down-regulates HYBID expression in skin fibroblasts, respectively^5,7^. We have also reported that HYBID is overexpressed in OA articular cartilage and synovium^11,12^, and disclosed that tumor necrosis factor-α (TNF-α) promotes HYBID expression in OA chondrocytes^11^, while interleukin-6 (IL-6), but not TNF-α, up-regulates the expression in OA synovial fibroblasts^12^, suggesting cell-type specific stimulation. Our data showed that HYBID expression is closely associated with HA depletion from OA articular cartilage and the increased rate of lower-molecular-weight HA (LMW-HA) to total HA in OA synovial fluid^11,12^ and suggested that HYBID may play an important role in the articular cartilage destruction through HA degradation in the cartilage and synovial fluid. However, there is an absence of information for the absolute expression levels of HYBID and TMEM2 in OA cartilage or the effect of IL-6 on HYBID expression in OA chondrocytes^11^. Although siRNA-mediated knockdown of HYBID results in disappearance of HA-degrading activity in OA chondrocytes and synovial fibroblasts^11,12^, effects of TMEM2 knockdown on HA-degrading activity are not studied in these cells. In addition, regulation of the TMEM2 expression by pro-inflammatory mediators in OA chondrocytes are as yet unknown.

In the present study, we examined the absolute expression levels of HYBID and TMEM2 in human OA cartilage tissue and investigated the effect of IL-6 on HYBID expression and various pro-inflammatory mediators on the TMEM2 expression in OA chondrocytes. In addition, HA-degrading activity of HYBID and TMEM2 was examined by siRNA-mediated knockdown experiments. Our study demonstrates that in contrast to TMEM2, HYBID is overexpressed, responsible for HA degradation, and up-regulated by IL-6 and TNF-α, i.e., more efficiently by IL-6 than TNF-α, in OA chondrocytes.

## Results

### Expression of HYBID and TMEM2 in OA cartilage

**Figure 1:**
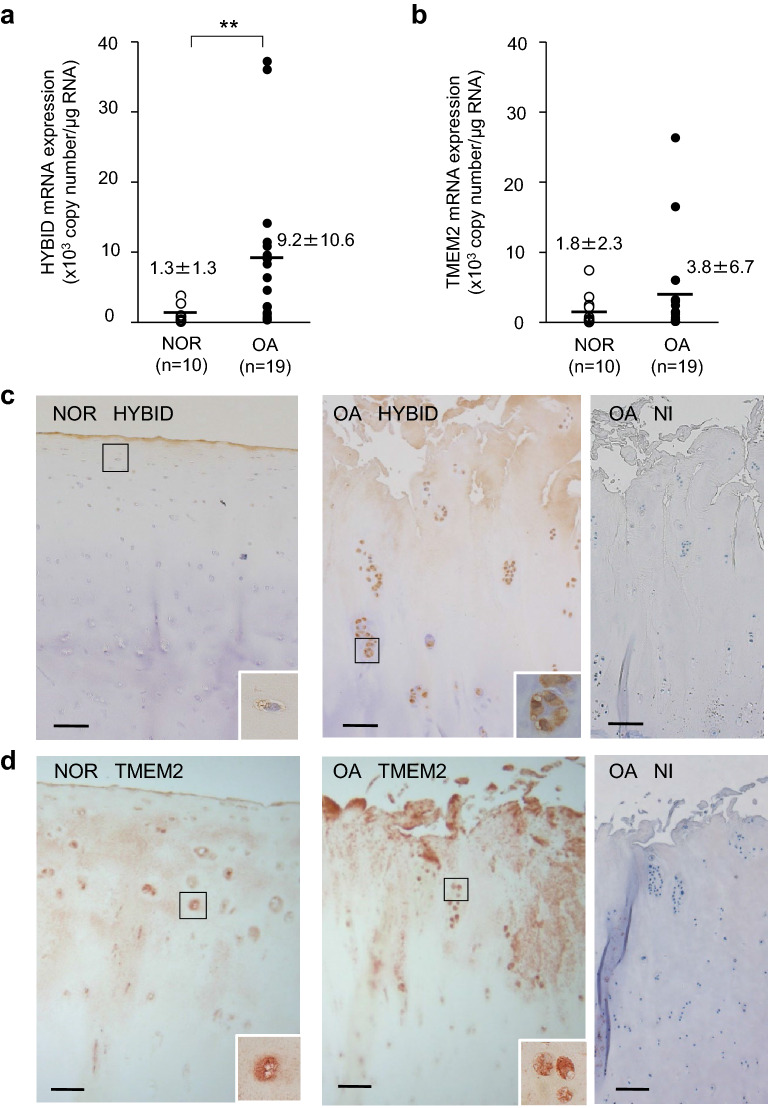
Expression of HYBID and TMEM2 and their tissue localization in human articular cartilage. (**a**) and (**b**) Copy numbers of HYBID and TMEM2 transcripts in control normal (NOR) and OA cartilage samples. The copy numbers were determined by quantitative real-time PCR assay with standard curves generated from reference plasmids. Symbols represent individual subjects, and horizontal lines indicate means. **, *P* < 0.01. (**c**) and (**d**) Immunohistochemistry for HYBID and TMEM2 in human cartilage tissue. Paraffin sections of the NOR and OA cartilage samples were stained with anti-HYBID antibody (HYBID), anti-TMEM2 antibody (TMEM2) or non-immune IgG (NI). Boxed areas are shown at higher magnification in the inset. Scale bars = 200 µm. Full-length images for the inset are presented in Supplementary Figure S1b.

We measured the mRNA copy numbers of HYBID and TMEM2 and found that HYBID expression level is significantly 7.1-fold higher in OA cartilage (9.2 ± 10.6 × 10^3^ copies per μg of total RNA) than in normal control cartilage (1.3 ± 1.3 × 10^3^ copies per μg of total RNA) (*P* = 0.003) (Fig. 1a). By contrast, the expression level of TMEM2 did not significantly differ between OA (3.8 ± 6.7 × 10^3^ copies per μg of total RNA) and control cartilage (1.8 ± 2.3 × 10^3^ copies per μg of total RNA) (*P* = 0.535) (Fig. 1b). By immunohistochemistry, HYBID was immunolocalized to many chondrocytes located in the superficial and intermediate zones of the OA articular cartilage, whereas only a few chondrocytes in the superficial zone of the normal cartilage showed weekly positive immunostaining (Fig. 1c), confirming the data of our previous study^11^. On the other hand, TMEM2 was immunostained by chondrocytes in the superficial and intermediate zones of both normal and OA articular cartilage (Fig. 1d). Only background staining was obtained by immunostaining with non-immune IgG in the normal and OA cartilage samples (Fig. 1c,d for OA cartilage, and Supplementary Figure S1a for normal cartilage). These data demonstrate that HYBID is overexpressed by chondrocytes in OA cartilage, while TMEM2 is constitutively expressed, and suggest that pro-inflammatory mediators present in OA joint may differentially regulate the expression of HYBID and TMEM2 in OA chondrocytes.

### Effects of pro-inflammatory mediators on HYBID and TMEM2 expression in OA chondrocytes

**Figure 2:**
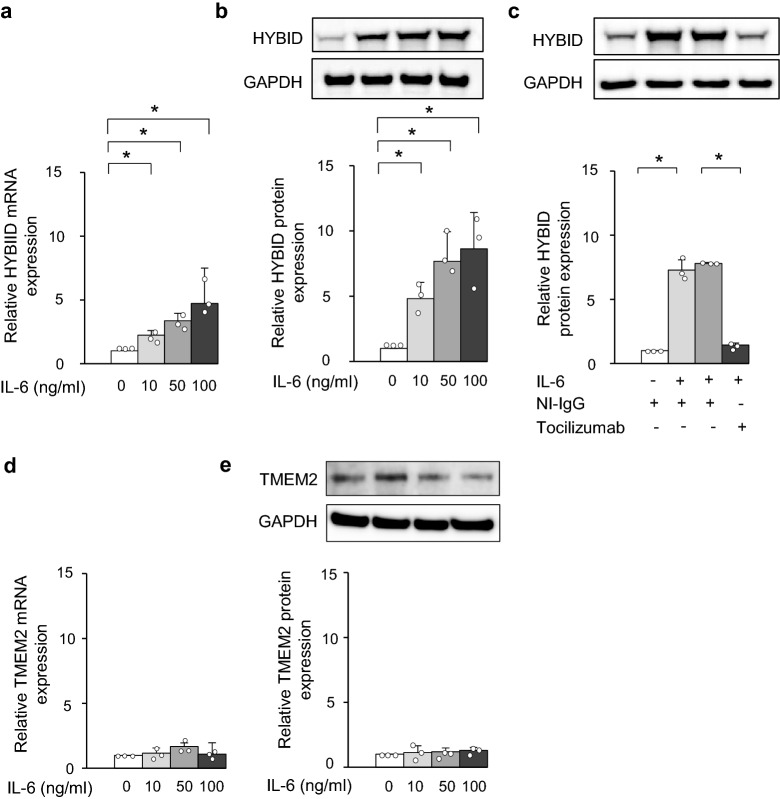
Increased HYBID expression by IL-6 in OA chondrocytes. (**a**) and (**b**) Effects of IL-6 on the mRNA and protein expression of HYBID in OA chondrocytes. OA chondrocytes at P2 were treated with IL-6 (0, 10, 50 or 100 ng/ml) and soluble IL-6 receptor (sIL-6R; 100 ng/ml). Cell lysates were harvested at 24 h after cultures for the HYBID mRNA expression and at 48 h for protein expression and subjected to quantitative real-time PCR using the ΔΔCt method (**a**) and immunoblotting with anti-HYBID antibody, followed by densitometric analysis (**b**). The average HYBID:GAPDH ratio in control OA chondrocytes treated with vehicle alone was set at 1. (**c**) Demonstration of IL-6-mediated HYBID protein overexpression in OA chondrocytes. OA chondrocytes at P2 were stimulated with IL-6 (0 or 100 ng/ml) and sIL-6R (100 ng/ml) in the presence of non-immune IgG (NI-IgG, 25 µg/ml) or anti-IL-6R antibody (tocilizumab, 25 µg/ml) for 48 h, and cell lysates were subjected to immunoblotting with anti-HYBID antibody, followed by densitometric analysis. (**d**) and (**e**) Effects of IL-6 on the mRNA and protein expression of TMEM2 in OA chondrocytes. OA chondrocytes at P2 were treated with IL-6 (0, 10, 50 or 100 ng/ml) and sIL-6R (100 ng/ml) for 24 h and 48 h, and TMEM2 expression was measured by quantitative real-time PCR using the ΔΔCt method (**d**) and immunoblotting, which was followed by densitometric analysis (**e**). The average TMEM2:GAPDH ratio in control OA chondrocytes treated with vehicle alone was set at 1. Values are expressed mean ± SD (n = 3). *, *P* < 0.05. The uncropped full-length gels can be found in Supplementary Figure S3.

Our previous study on HYBID expression in OA chondrocytes showed that only TNF-α up-regulates HYBID expression of eight pro-inflammatory factors^11^. Subsequently, we showed that IL-6 is a stimulator of HYBID expression in OA synovial fibroblasts^12^. Therefore, we tested whether IL-6 stimulates OA chondrocytes and demonstrated that IL-6 significantly promotes HYBID mRNA expression in a dose dependent manner (Fig. 2a). Immunoblotting analysis supported the stimulative effect of IL-6 on HYBID expression (Fig. 2b), and this stimulation was abrogated by treating them with anti-IL-6R antibody (tocilizumab) (Fig. 2c), indicating that the stimulative effect is due to IL-6.

We also examined effects of IL-6, IL-1α, IL-8, TNF-α, vascular endothelial growth factor_165_ (VEGF), basic fibroblast growth factor (bFGF), prostaglandin E2 (PGE2), insulin-like growth factor 1 (IGF-1), histamine and TGF-β on the TMEM2 expression in OA chondrocytes, since no information was available for their effects. The results indicated absence of up- or down-regulation of the TMEM2 mRNA expression by these factors (Fig. 2d for IL-6 and Supplementary Figure S2 for other factors). Also, immunoblotting analysis confirmed the data of no changes in TMEM2 expression by IL-6 treatment (Fig. 2e).

### Involvement of HYBID, but not TMEM2, in HA degradation by OA chondrocytes

**Figure 3:**
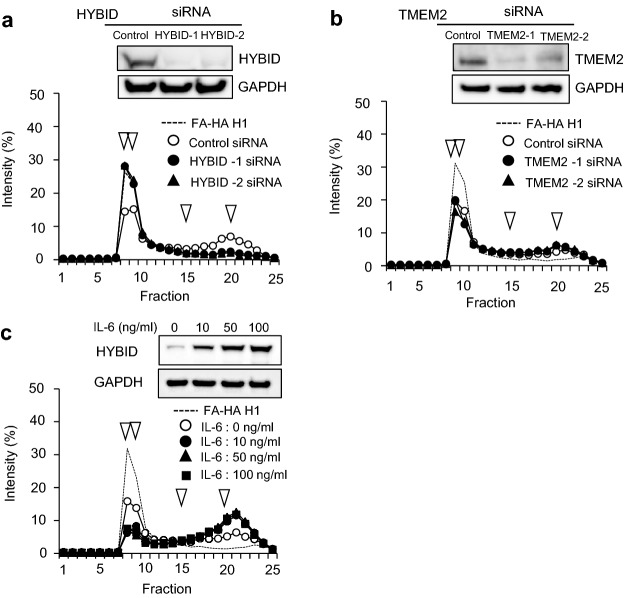
HYBID-dependent HA-degrading activity in OA chondrocytes and increased HA-degrading activity in IL-6-treated OA chondrocytes. (**a**) Abrogation of HA-degrading activity by siRNA-mediated knockdown HYBID expression. OA chondrocytes transfected with two-different siRNAs for HYBID (HYBID-1 and HYBID-2) or non-silencing RNA (Control) were cultured with HMW-HA (10 μg/ml FA-HA H1) for 48 h, and HA fragments in the culture media were analyzed by size-exclusion chromatography. The protein expression of HYBID and GAPDH (a loading control) in the transfected cells was examined by immunoblotting. (**b**) No inhibition of HA-degrading activity by siRNA-mediated knockdown for TMEM2 expression. OA chondrocytes transfected with two different siRNAs for TMEM2 (TMEM2-1 and TMEM2-2) or non-silencing RNA (Control) were cultured for 48 h, and HA-degrading activity was determined as described above. The protein expression of TMEM2 and GAPDH in the transfected cells was examined by immunoblotting. (**c**) Enhanced HA-degrading activity in OA chondrocytes by treating with IL-6. OA chondrocytes were stimulated with IL-6 (0, 10, 50 and 100 ng/ml) in the presence of sIL-6R (100 ng/ml) and cultured with FA-HA H1 for 48 h. HA-degrading activity was determined as described above. Increased HA-degrading activity appears to be saturated by treatment with 10 ng/ml IL-6 in this assay. Arrowheads indicate elution peaks of FA-HA species with 1,562, 907, 197, or 56 kDa from left to right. The uncropped full-length gels are presented in Supplementary Figure S4.

The expression of HYBID and TMEM2 was knocked down by siRNAs in OA chondrocytes and changes in their HA-degrading activity were determined by culturing these cells in media containing HMW-HA, i.e., fluoresceinamine-labeled HA (FA-HA) H1 and applying the media to size-exclusion chromatography. As shown in Fig. 3a, siRNAs targeting HYBID (HYBID-1 and HYBID-2 siRNAs) almost completely suppressed the HYBID protein expression and HA-degrading activity disappeared. By contrast, HA-degrading activity of the chondrocytes was not changed by knockdown of the TMEM2 expression (Fig. 3b). In addition, when OA chondrocytes were treated with IL-6, HYBID expression was up-regulated and HA-degrading activity was increased in chondrocytes (Fig. 3c).

### Increased expression of HYBID by TNF-α and/or IL-6 in OA chondrocytes

**Figure 4:**
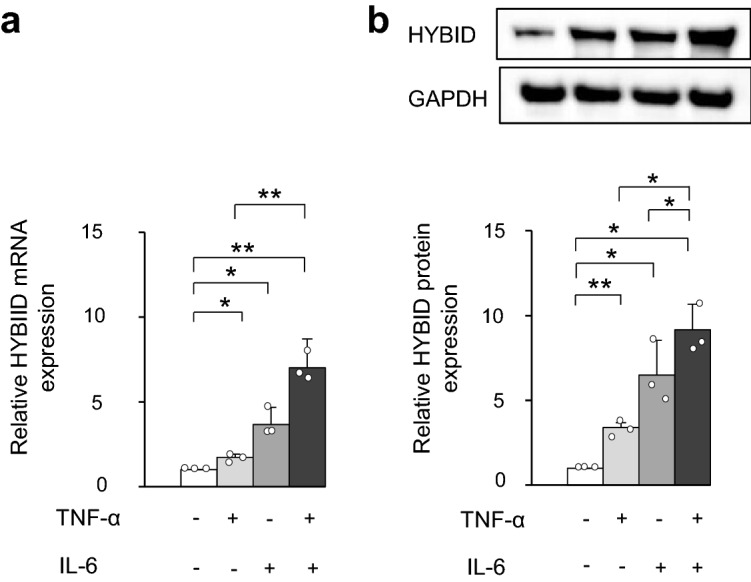
Additive effect of the HYBID expression by TNF-α and IL-6 in OA chondrocytes. (**a**) and (**b**) Effects of TNF-α and IL-6 on the mRNA and protein expression of HYBID in OA chondrocytes, respectively. OA chondrocytes at P2 were treated with TNF-α (0 or 10 ng/ml) and/or IL-6 (0, 100 ng/ml) in the presence of sIL-6R (100 ng/ml) for 24 h and 48 h, and cell lysates were subjected to quantitative real-time PCR for HYBID expression using the ΔΔCt method (**a**) and immunoblotting with anti-HYBID antibody, followed by densitometric analysis (**b**). The average HYBID:GAPDH ratio in control OA chondrocytes treated with vehicle was set at 1. Values are expressed mean ± SD (n = 3). *, *P* < 0.05; **, *P* < 0.01. The uncropped full-length gels are presented in Supplementary Figure S5.

To examine the effect of TNF-α and IL-6 on the HYBID expression in OA chondrocytes, we treated OA chondrocytes with TNF-α and/or IL-6. As shown in Fig. 4a, the mRNA expression level of HYBID was significantly increased by treatment with TNF-α (1.7 ± 0.2) or IL-6 (3.7 ± 1.0) as compared to the control group without treatment, and up-regulated by combined treatment with TNF-α and IL-6 (7.0 ± 0.4). Accordingly, the HYBID protein expression was significantly increased by treatment with TNF-α (3.4 ± 0.3), IL-6 (6.5 ± 2.1), or TNF-α and IL-6 (9.2 ± 1.5) (Fig. 4b). The mRNA and protein expression levels in chondrocytes treated with both TNF-α and IL-6 were significantly higher than those treated with TNF-α or IL-6 alone (*P* < 0.01 or *P* < 0.05), although the mRNA level was not significantly different between IL-6 and combined treatment groups (*P* = 0.051) (Fig. 4). Since the levels after combined treatment were near to the sum of their individual effects, it is plausible that the effect is additive.

### Involvement of IL-6 in HYBID expression under stimulation with IL-1α in OA chondrocytes

**Figure 5:**
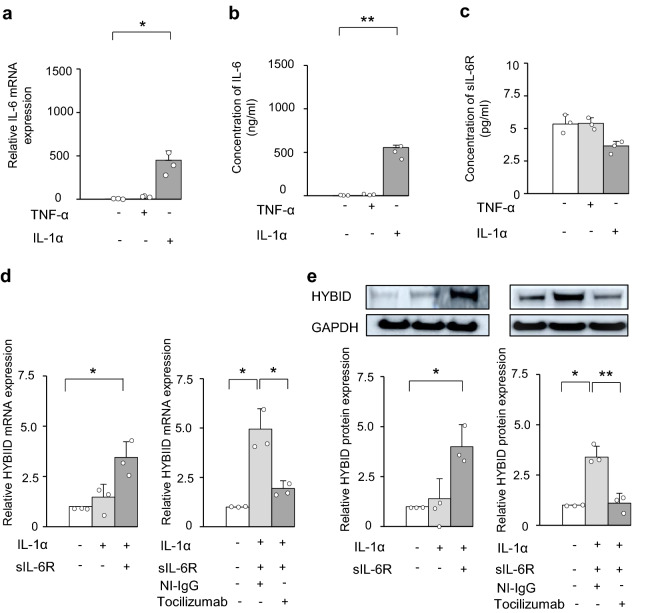
Expression of IL-6 and sIL-6R in OA chondrocytes treated with TNF-α or IL-1α and involvement of IL-6 in IL-1α-stimulated HYBID overexpression. (**a**) and (**b**) Overexpression of IL-6 in OA chondrocytes treated with IL-1α. OA chondrocytes at P2 were treated with TNF-α (0 or 10 ng/ml) or IL-1α (0 or 10 ng/ml) for 24 h, and mRNA isolated from cell lysates was subjected to quantitative real-time PCR for IL-6 using the ΔΔCt method (**a**). Concentrations of IL-6 produced into culture media by OA chondrocytes treated with TNF-α (0 or 10 ng/ml) or IL-1α (0 or 10 ng/ml) for 48 h were measured by ELISA (**b**). The average HYBID:GAPDH ratio in control OA chondrocytes treated with vehicle was set at 1. (**c**) Concentration of sIL-6R in culture media of OA chondrocytes treated with IL-1α or TNF-α. OA chondrocytes were treated with IL-1α (0 or 10 ng/ml) or TNF-α (0 or 10 ng/ml) for 24 h and culture media were subjected to ELISA for sIL-6R. (**d**) and (**e**) IL-6-mediated HYBID overexpression in OA chondrocytes stimulated with IL-1α. OA chondrocytes were treated with IL-1α (0 or 10 ng/ml) in the presence or absence of sIL-6R (100 ng/ml) for 24 h and 48 h, and the mRNA and protein expression of HYBID was analyzed by quantitative real-time PCR using the ΔΔCt method (**d**) and immunoblotting with anti-HYBID antibody (**e**). To examine the involvement of IL-6 in IL-1α-stimulated HYBID expression, the expression was analyzed by treating the IL-1α (10 ng/ml) and sIL-6R (100 ng/ml)-stimulated chondrocytes with non-immune IgG (NI-IgG, 25 µg/ml) or anti-IL-6R antibody (tocilizumab, 25 µg/ml). Values are expressed mean ± SD (n = 3). *, *P* < 0.05; **, *P* < 0.01. The uncropped full-length gels are presented in Supplementary Figure S6.

We reported that IL-1α alone has no stimulative effect on HYBID expression^11^. However, since previous study showed that IL-1β and TNF-α increase the production levels of IL-6 in human articular chondrocytes^13^, we tested the possibility of the involvement of IL-6 in HYBID expression in OA chondrocytes under stimulation with IL1-α or TNF-α. As shown in Fig. 5a,b, IL-1α remarkably increased the mRNA and protein levels of IL-6, although no such effect was observed with TNF-α. Under these conditions, the production levels of soluble IL-6 receptor (sIL-6R) were not significantly changed (Fig. 5c). We then examined HYBID expression by IL-1α in the presence of sIL-6R and found that HYBID mRNA and protein expression is significantly increased by treatment with IL-1α in the presence of sIL-6R (Fig. 5d,e). Importantly, HYBID expression by treatment with IL-1α and sIL-6R was abrogated by treatment with anti-IL-6R antibody (tocilizumab), but not non-immune IgG (Fig. 5d,e).

## Discussion

In the present study, we have demonstrated for the first time that absolute expression level of HYBID is significantly enhanced sevenfold in OA cartilage compared to control normal cartilage and is consistent with our previous results that HYBID is fourfold overexpressed in OA cartilage to normal cartilage by quantitative real-time PCR analysis^11^. Additionally, the current study has indicated that the absolute expression level of TMEM2 is not significantly different between OA and normal cartilage, and the HYBID level in OA cartilage is 2.4-fold higher than that of TMEM2. The similar expression profiles of HYBID and TMEM2 are observed in OA and normal synovial tissues^12^. Accordingly, these data demonstrate the contrast in the expression patterns between HYBID and TMEM2, and could suggest that HYBID expression in OA cartilage and synovial tissues is up-regulated by local factors such as pro-inflammatory mediators present in OA joint.

Our previous study showed that TNF-α promotes HYBID expression in OA chondrocytes^11^. In the present study, we have confirmed the TNF-α-mediated stimulation of HYBID expression in OA chondrocytes, but further demonstrated that IL-6 stimulates OA chondrocytes to overexpress HYBID twofold more than TNF-α. The current study has also provided the first evidence that HYBID mRNA and protein expression is additively up-regulated by combined treatment with TNF-α and IL-6. We previously reported that TNF-α has no stimulative effect on HYBID expression in OA synovial fibroblasts and suggested that regulation of the HYBID expression is cell-type specific^12^. Cellular and molecular mechanisms on the different TNF-α reactivity to OA chondrocytes and synovial fibroblasts remain unknown at the present time. However, TNF-α-induced activity is dependent on expression of the TNF-receptor (TNF-R), which is composed of two isoforms, p55 TNF-R1 and p75 TNF-R2^14^, and cell-surface expression of these receptors is regulated by proteolytic shedding of the ectodomain^15^. Both TNF-R1 and TNF-R2 are known to be readily shed from OA synovial fibroblasts during culture and TNF-R2 shedding is increased by TNF-α treatment^16^. In contrast, shedding of TNF-R1 and TNF-R2 is reportedly only minimal in cultured OA chondrocytes^17^. Therefore, it seems likely that negligible effect of TNF-α on cultured OA synovial fibroblasts may be due to the reduced expression of cell-surface TNF-Rs, as discussed in the previous study^18^.

Our previous^12^ and present studies indicated that TMEM2 is constitutively expressed in synovial and cartilage tissues without showing significant differences between normal and OA groups. However, TMEM2 is reportedly up-regulated in various human cancer cell lines^10^ and in embryonic tissues of mice^9^ and zebrafish^19^. Since SOX4, which is overexpressed in breast carcinoma cells, up-regulates the TMEM2 expression by its direct binding to the promoter region^20^, the increased expression of TMEM2 in carcer cell lines may be explained by the SOX4-mediated pathway^20^. SOX4 is also overexpressed in various embryonic tissues including the endocardial cushions and ridges during development and organogenesis in mice, and Sox4-deficient mice show the developmental heart abnormality, i.e., a common arterial trunk defect^21^, which resembles a developmental phenotype of zebrafish tmem2 mutants^19^. Thus, SOX4 may play a key role in the enhanced expression of Tmem2 in the developing mouse^9^ and zebrafish tissues^19^. However, the regulatory mechanism of the constitutive expression of TMEM2 in human adult tissues including synovium and cartilage remains to be determined in future studies.

Previously, the HA-degrading activity of TMEM2 was shown in a solution assay of FA-HA that was incubated with cultured 293T cells transiently transfected with TMEM2-expression vectors, their cell membrane fractions and recombinant protein of the extracellular domain of TMEM2^9^. In addition, pericellular HA-degrading activity of cancer cell lines was recently shown by in situ FA-HA degradation assay, in which the activity was detected as fluorescence-negative areas after culturing the cells on coverslips coated with FA-HA^10^. However, the HA-degrading activity in primary cells isolated from human tissues such as OA chondrocytes and synovial fibroblasts has not been verified. In the present study, we investigated the HA-degrading activity in a solution assay and found that siRNA-mediated knockdown of TMEM2 in OA chondrocytes showed no changes in the activity, whereas the knockdown of HYBID and IL-6-mediated HYBID overexpression vanished and increased the activity, respectively. Therefore, our data demonstrate, to the best of our knowledge, for the first time that the HA-degrading activity of OA chondrocytes is due to HYBID, but not TMEM2.

Differential roles of HYBID and TMEM2 in normal and OA cartilage remain to be clarified. However, our recent study on the experimental knee OA models using Hybid-deficient mice demonstrated that cartilage destruction and osteophyte formation are suppressed in Hybid-deficient mice and suggested that Hybid plays a key role in the progression of mechanically induced knee OA by HA degradation in joint tissues including the articular cartilage and synovium^22^, and could be a potential therapeutic target on the onset of OA^23^. In addition, a very recent study by Deroyer et al. reported that HYBID (CEMIP, KIAA1199) overexpressed by OA synovial tissue is implicated for synovial inflammation and hyperplasia^24^. On the other hand, TMEM2 is reported to contribute to ER stress resistance through binding of LMW-HA fragments generated by the action of TMEM2 to cell-surface receptor CD44^25^ and promote cell attachment and migration by pericellular digestion of HMW-HA, which acts as a gel-like anti-adhesive barrier to cells^10^. Thus, it is plausible to speculate that constitutively expressed TMEM2 in synovium and cartilage may be involved in physiological turnover of HA at cell surfaces in these tissues to regulate attachment and migration of synovial cells and chondrocytes.

IL-1 is overexpressed in OA cartilage^26^, and IL-1 and TNF-α are over-produced in OA synovial tissue^27,28^. Interestingly, a previous study showed that both IL-1 and TNF-α stimulate human chondrocytes to overexpress IL-6^13^. In the present study, we have demonstrated that IL-1α, but not TNF-α, is a strong inducer for IL-6 in OA chondrocytes, resulting in over-production of IL-6 at a concentration of ~ 500 ng/ml after a 24 h-culture under IL-1α treatment. Importantly, our study has demonstrated that the HYBID expression is enhanced by IL-1α treatment only when sIL-6R is supplemented to the culture, since sIL-6R production was not increased by IL-1α treatment. These data suggest the possibility that IL-1 may be implicated for HYBID overexpression via the IL-6 pathway.

Clinically, overloaded mechanical stress to the articular cartilage is one of the most important factors to cause the pathological changes in OA cartilage^29^, in which the HA-aggrecan network is initially degraded and disappears, followed by disruption of the collagen fibrils^1,2^. Increased mechanical stress is known to stimulate cultured chondrocytes to induce the expression of TNF-α and IL-6^30,31^ and the present study has demonstrated the TNF-α- and/or IL-6-mediated overexpression of HYBID in OA chondrocytes. Thus, both cytokines seem to be linked to the initial damage of the articular cartilage through HYBID-mediated HA degradation. In addition, LMW-HA fragments are known to activate Toll-like receptor 4 (TLR4)^32^ and induce the IL-1β, IL-6 and TNF-α expression in human chondrocytes^33^. Altogether, these data suggest a mechanical stress-induced vicious cycle for OA cartilage destruction via HYBID-mediated HA degradation. OA joints are commonly accompanied by synovitis, which may be induced by ingestion of degraded materials of articular cartilage by synovial cells^34^. Once OA synovitis starts, IL-1, TNF-α and IL-6 are over-produced and secreted to synovial fluid through activation of TLR4 by synovial cells^35^. Therefore, these cytokines may not only promote the HA degradation via HYBID overexpression in the articular cartilage and synovial fluid but also accelerate the vicious cycle in OA joint^12^.

In summary, we have provided the first evidence that the absolute expression level of HYBID is increased in OA cartilage compared to normal cartilage, and that HYBID expression is enhanced more efficiently by IL-6 than TNF-α and additively up-regulated by their combined treatment in OA chondrocytes. These data show a sharp contrast to the constitutive expression of TMEM2. Since our previous^12^ and present studies have demonstrated that IL-6 is a strong stimulator of HYBID expression in OA synovial fibroblasts and chondrocytes, IL-6 may be a molecular target in OA patients. Therapeutic strategies may include application of blockade of IL-6 or IL-6R and development of inhibitors to HYBID activity and/or molecules to selectively suppress the expression, although further studies are necessary. Accumulated lines of evidence have disclosed the implications of HYBID for various pathological conditions: HYBID is overexpressed and suggested to contribute to HA degradation in several inflammatory conditions, which include rheumatoid arthritis^36^, Crohn’s disease^37^, and photoaged skin^38,39^. In addition, HYBID up-regulation in cancer tissues may play key roles in tumor cell survival^40^, tumor growth^41^, migration and invasion^42^, and brain metastasis^43^, although the mechanisms involved in these biological processes are not completely understood. Therefore, beyond OA, the data in the current study on the expression and regulation of HYBID and TMEM2 would provide the cross-disciplinary impact on the research fields of inflammatory and neoplastic diseases.

## Methods

### Clinical samples and histology

Non-osteophytic articular cartilage samples with macroscopic OA changes were obtained at arthroplasty from joints of the patients with knee OA (n = 19; mean age 68 ± 15, range 43–88 years), which was diagnosed according to the criteria of the American College of Rheumatology^44^. Control cartilage samples showing a normal appearance were obtained from hip joints of patients with femoral neck fracture (n = 10; mean age 78 ± 10, range 59–88 years). These cartilage samples were minced, freeze-milled using CoolMill (Toyobo, Life Science, Tokyo, Japan), and then subjected to RNA extraction. For some cases, tissue slices obtained from OA and normal cartilage were fixed with 4% paraformaldehyde, decalcified with 10% EDTA (pH 7.4), and embedded in paraffin. For the experimental use of the surgical samples, informed consent was obtained from the patients according to the hospital ethics guidelines. The study protocols complied with the principles outlined in the Declaration of Helsinki and were approved by the Ethical Committee Review Board in Juntendo University (No 15-074).

### Quantification of mRNA copy numbers

Total RNA (2 μg) isolated by RNeasy Mini Kit (Qiagen, Hilden, Germany) was utilized to synthesize cDNA by using the ReverTraAce qPCR RT Master Mix (Toyobo, Osaka, Japan). Quantitative real-time PCR was performed by using the THUNDERBIRD SYBR qPCR Mix (Toyobo, Osaka, Japan) on a QuantStudio3 (Applied Biosystems, Foster City, CA) according to our previous methods^12^. The primers were as follows: for HYBID 5′-TCACAGAGGACTCCTACCCG-3′ (forward) and 5′-ATTGGCCATCCAGAAGGTGG-3′ (reverse); and for TMEM2 5′-TTACGGCTTTCAGGGTGGTC-3′ (forward) and 5′-TTGGGAACGTCCTGTTCCTG -3′ (reverse)^12^. Absolute quantification of mRNA copy numbers of HYBID and TMEM2 was performed using standard curve method as we previously described^12^. Purified plasmids containing the coding sequences of human HYBID (pcDNA3.1(-)-HYBID)^5^ and human TMEM2 (RC224793; Origene, Rockville, MD) were digested with restriction enzymes to isolate the inserts from the plasmids. Concentrations of the plasmid and insert were determined using the QuantiFluor dsDNA System (Promega, Madison, WI), and copy numbers were calculated according to the following formula: Copy number (g/molecule) = (base pairs size of double-stranded plasmid containing the insert) x (330 daltons × 2 nucleotides/base pairs) ÷ (Avogadro’s number 6.023 × 10^23^ molecules/mole). Standard curves of plasmid DNA containing the coding regions of HYBID and TMEM2 were generated with Ct values obtained from quantitative real-time PCR of various copy numbers of the plasmids^12^. The copy numbers of HYBID and TMEM2 in the cartilage samples were calculated by relating the PCR signal (Ct value) of each cartilage tissue sample to the standard curves.

### Immunohistochemistry

For immunostaining of HYBID, paraffin sections were subjected to antigen retrieval by boiling in 10 mM citrate buffer (pH 6.0) for 10 min, and then treated with 3% H_2_O_2_ and BLOCK ACE (DS Pharma Biomedical, Osaka, Japan) to block peroxidase and nonspecific reactions, respectively^11,12^. They were immunostained with rabbit anti-HYBID (KIAA1199) antibody (SAB2105467; Sigma-Aldrich, St Louis, MO) according to our previous methods^12^. For TMEM2 immunostaining, antigen retrieval was carried out by treating with 100 mM 2-mercaptoethanol in 20 mM Tris–HCl buffer (pH 9.0) for 60 min at room temperature, followed by reaction with 100 mM iodoacetamide in 100 mM Tris–HCl buffer (pH 9.0) for 15 min^45^. After blocking peroxidase and nonspecific reactions, they were incubated with rabbit anti-TMEM2 antibody (SAB2105088; Sigma-Aldrich). Then, incubation with biotinylated antibody against rabbit IgG was followed by the ABC methods (Vector Laboratories, Burlingame, CA). After color development with 3-amino-9-ethylcarbazole chromogen (Vector Laboratories), counterstaining was performed with hematoxylin^12^.

### Cultures of OA chondrocytes

Chondrocytes were isolated from knee OA cartilage by enzymatic dissociation and cultured in Dulbecco’s modified Eagle’s medium/Ham’s F-12 (DMEM/F-12) (Sigma-Aldrich) containing 10% fetal bovine serum (FBS) and 25 µg/ml ascorbic acid^46^. They were used for experiments at passage numbers 1–3 (P1-3).

### Stimulation of OA chondrocytes with factors

Serum-starved OA chondrocytes at P2 were treated with IL-6 (R&D Systems, Minneapolis, MN) plus sIL-6R (R&D Systems), IL-8 (R&D Systems), TNF-α (R&D Systems), IL-1α (R&D Systems), VEGF (R&D Systems), bFGF (Sigma-Aldrich), PGE2 (Sigma-Aldrich), IGF-1 (Sigma-Aldrich), histamine (Wako, Osaka, Japan) and TGF-β1 (R&D Systems), or vehicle alone in DMEM/F-12 containing 1% FBS for 24 h. The expression levels of HYBID and TMEM2 were determined by normalizing to glyceraldehyde-3-phosphate dehydrogenase (GAPDH) using the THUNDERBIRD SYBR qPCR Mix (Toyobo) according to the ΔΔCt method^47^. The primers were as follows: for HYBID 5′-AGGGAAGCAGGTCAGAGTGA-3′ (forward) and 5′-TCTCGGCTACAGACCCAGAG-3′ (reverse); for TMEM2 5′-ACTTGGTGGCTGGCATGTTC-3′ (forward) and 5′-CATGAGCTGGGCCTGAGTTG-3′ (reverse); and for GAPDH 5′-GCACCGTCAAGGCTGAGAAC-3′ (forward) and 5′-TGGTGAAGACGCCAGTGGA-3′ (reverse)^12^. Similarly, HYBID expression was also examined in OA chondrocytes treated with TNF-α and IL-6 in the presence of sIL-6R. Protein expression of HYBID was analyzed in chondrocytes stimulated with the cytokines for 48 h by immunoblotting, as described below.

### Immunoblotting

Cell lysates of OA chondrocytes, which were treated with the cytokines or siRNAs for HYBID or TMEM2 (see below), were harvested with 2× sodium dodecyl sulfate–polyacrylamide gel electrophoresis (SDS-PAGE) sample buffer containing 2-mercaptoethanol. The proteins resolved on the gels by SDS-PAGE were transferred onto PVDF membranes, and they were reacted with anti-HYBID antibody (Proteintech, Rosemont, IL), anti-TMEM2 antibody (SAB2105088; Sigma-Aldrich) and anti-GAPDH antibody (as a loading control) (ab125247, Abcam), followed by incubation with Envision + System HRP-labeled polymer anti-rabbit IgG (Dako, Glostrup, Denmark)^12^. The intensity of the immunoreactive bands detected by chemiluminescence imaging system using Amersham Imager 680 (GE Healthcare Life Sciences, Tokyo, Japan) was quantified by densitometric analysis using ImageJ software (http//rsb.info.nih.gov/ij/).

### RNA interference for HYBID and TMEM2

Two different siRNAs designed to target HYBID or TMEM2 and non-silencing control RNAs were purchased from ThermoFisher Scientific (Waltham, MA). OA chondrocytes were transfected with these siRNAs by electroporation using a Nucleofector kit (Amaxa, Gaithersburg, MD) according to the manufacturer’s protocol. The transfected chondrocytes were used for the experiment at 48 h after transfection. Knockdown of the expression was confirmed by immunoblotting with anti-HYBID antibody, anti-TMEM2 antibody and anti-GAPDH antibody^12^.

### Determination of HA-degrading activity

Cellular depolymerization of HMW-HA was assessed by culturing confluent siRNA-transfected or IL-6-treated OA chondrocytes in DMEM/F-12 medium containing 1% FBS and 10 μg/ml FA-HA H1 (1,562 kDa; average molecular size) and by applying the media harvested after 48 h to a Sepharose CL-2B column (GE Healthcare, Tokyo, Japan) equilibrated with 0.5 M NaCl in distilled water according to our previous methods^5^. Calibration was carried out using the FA-HA species including H1 (1,562 kDa), M1 (907 kDa), L1 (197 kDa) and S1 (56 kDa), all of which were purchased from PG Research (Tokyo, Japan)^12^.

### Expression of IL-6 and measurement of IL-6 and sIL-6R in culture media of OA chondrocytes

Serum-starved OA chondrocytes at P2 were treated with IL-1α (0 or 10 ng/ml) or TNF-α (0 or 10 ng/ml) for 24 h in DMEM/F-12 medium containing 1% FBS, and the mRNA expression level of IL-6 was determined by normalizing to GAPDH using quantitative real-time PCR according to the ΔΔCt method. The primers were as follows: for IL-6 5′-AAGCCAGAGCTGTGCAGATGAGTA-3′ (forward) and 5′-TGTCCTGCAGCCACTGGTTC-3′ (reverse); and for GAPDH 5′-GCACCGTCAAGGCTGAGAAC-3′ (forward) and 5′-TGGTGAAGACGCCAGTGGA-3′ (reverse). The concentration of IL-6 and sIL-6R in culture media of OA chondrocytes was measured using the ELISA kits (R&D Systems) according to the manufacturer`s protocols. The culture media were prepared by treating OA chondrocytes with IL-1α (0 or 10 ng/ml) or TNF-α (0 or 10 ng/ml) for 24 h in DMEM/F-12 medium containing 1% FBS, and then subjected to the assays.

### Effects of tocilizumab on OA chondrocytes

OA chondrocytes were treated with humanized anti-IL-6R antibody (tocilizumab; 25 µg/ml) (Actemra; Chugai Pharmaceutical Co., LTD, Tokyo, Japan) or non-immune human IgG (R&D Systems; 25 µg/ml) in DMEM/F-12 containing 1% FBS for 1 h prior to stimulation with IL-6 (0 or 100 ng/ml) or IL-1α (0 or 10 ng/ml) in the presence of sIL-6R (100 ng/ml) for 48 h, and then subjected to immunoblotting with anti-HYBID antibody according to our previous methods^12^.

### Statistical analysis

All data were analyzed using IBM SPSS Statistics 21.0 software program and expressed as the mean ± SD. For comparisons of the HYBID and TMEM2 expression levels between normal and OA cartilage samples, the Mann–Whitney U test was used. Comparisons involving more than 3 groups in the experiments using knee OA chondrocytes were performed by the one-way analysis of variance (ANOVA), followed by the Bonferroni test. Statistical significance was determined by the Student’s t test (*P* < 0.05).

## Supplementary Information

Supplementary Information.

## Data Availability

All data generated or analyzed during this study are included in Supplementary Information files.

## Abbreviations

ADAMTS: A disintegrin and metalloproteinase with thrombospondin motifs
bFGF: Basic fibroblast growth factor
CEMIP: Cell migration-inducing protein
ELISA: Enzyme-linked immunosorbent assay
FA-HA: Fluoreceinamine-labeled hyaluronan
FBS: Fetal bovine serum
GAPDH: Glyceraldehyde-3-phosphate dehydrogenase
HA: Hyaluronan
HMW-HA: High-molecular-weight hyaluronan
HYAL: Hyaluronidase
HYBID: Hyaluronan-binding protein involved in hyaluronan depolymerization
IGF-1: Insulin-like growth factor-1
IL-1: Interleukin-1
IL-6: Interleukin-6
IL-8: Interleukin-8
LMW-HA: Lower-molecular-weight hyaluronan
MMP: Matrix metalloproteinase
OA: Osteoarthritis
PBS: Phosphate buffered saline
PGE2: Prostaglandin E2
RT-PCR: Reverse transcription-polymerase chain reaction
SDS-PAGE: Sodium dodecyl sulfate–polyacrylamide gel electrophoresis
sIL-6R: Soluble interleukin-6 receptor
TGF-β: Transforming growth factor-β
TLR4: Toll-like receptor 4
TMEM2: Transmembrane protein 2
TNF-α: Tumor necrosis factor-α
TNF-R: Tumor necrosis factor-receptor
VEGF: Vascular endothelial growth factor
