# Supplementary material for: Expression and regulation of recently discovered hyaluronidases, HYBID and TMEM2, in chondrocytes from knee osteoarthritic cartilage
Source: Sci Rep. 2022 Oct 14;12:17242. doi: 10.1038/s41598-022-22230-z (PMC9568606; doi:10.1038/s41598-022-22230-z)
Supplement: Supplementary file 1 — Supplementary Information. [file 41598_2022_22230_MOESM1_ESM.pdf]

Supplementary Figure S1

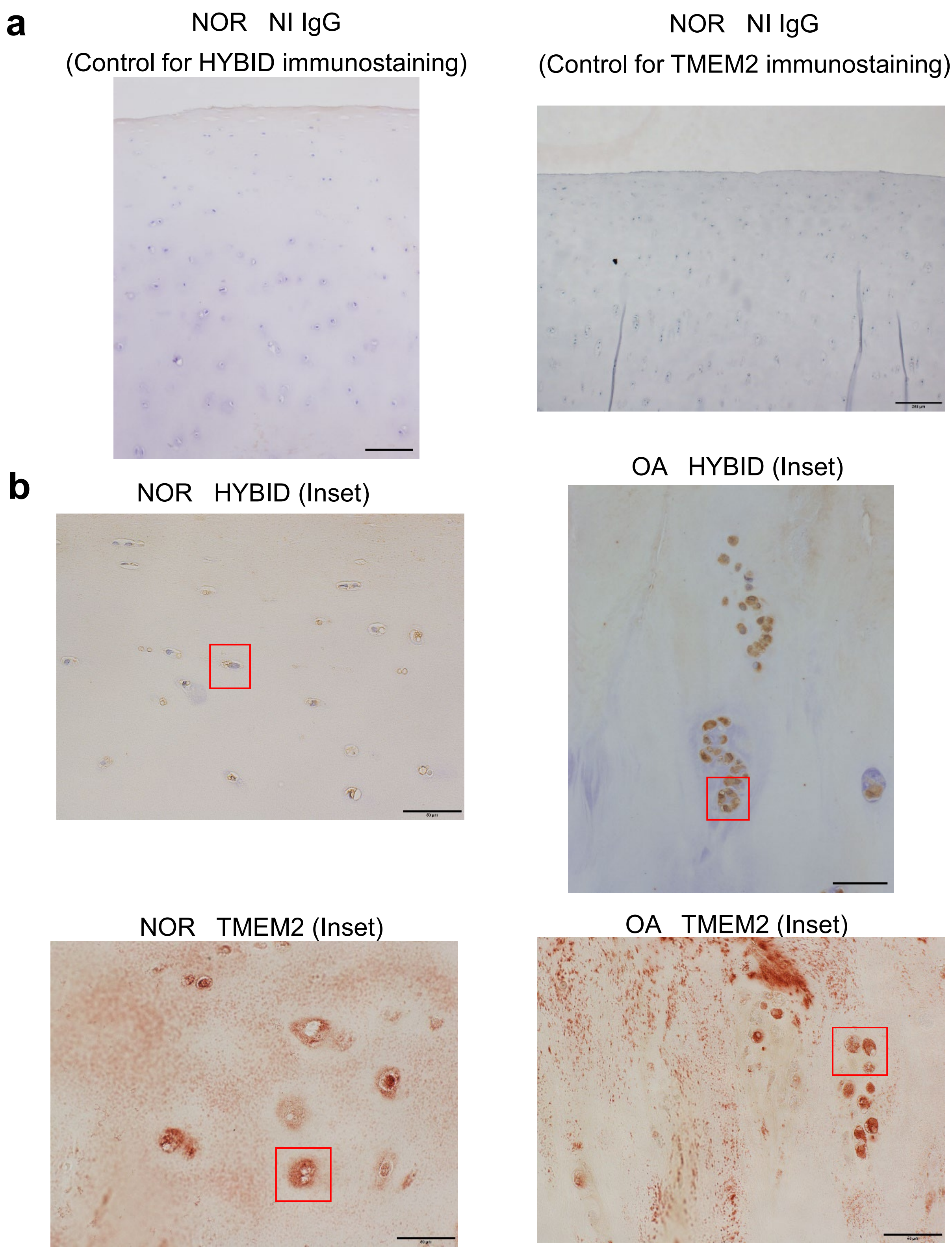

Supplementary Figure S1. (a) Immunostaining of normal cartilage (NOR) with rabbit non-immune (NI) IgG. Paraffin sections were immunostained with NI IgG after antigen retrieval and blocking nonspecific reactions. Note only background staining in the cartilage. Bar, 200 μm. **(b)** Original full-length images for insets in Figure 1c and 1d. Boxed areas were used for insets. Bar, 50 μm.

Supplementary Figure S2

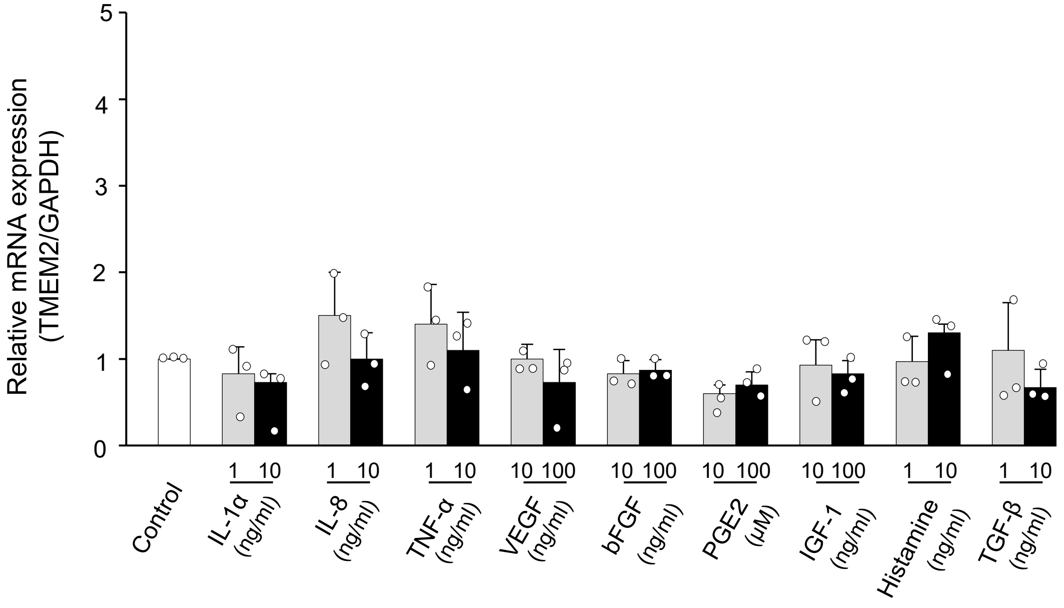

Supplementary Figure S2. Quantitative real-time PCR analysis of TMEM2 expression in OA chondrocytes after treatment with the factors. OA chondrocytes at P2 were treated with IL-1α (1 and 10 ng/ml), IL-8 (1 and 10 ng/ml), TNF-α (1 and 10 ng/ml) VEGF (10 and 100 ng/ml), bFGF (10 and 100 ng/ml), PGE2 (10 and 100 μM), IGF-1 (10 and 100 ng/ml), histamine (1 and 10 ng/ml) or TGF-β (1 and 10 ng/ml) for 24 h. Relative expression of TMEM2 was determined by normalizing the samples to the expression level of GAPDH transcripts using the ΔΔCt method. The mean TMEM2:GAPDH ratio in control chondrocytes treated with vehicle alone was set at 1. Note negligible changes in the expression by treatment with the factors.

Supplementary Figure S3

Full-length gels for **Figure 2b** (HYBID and GAPDH)

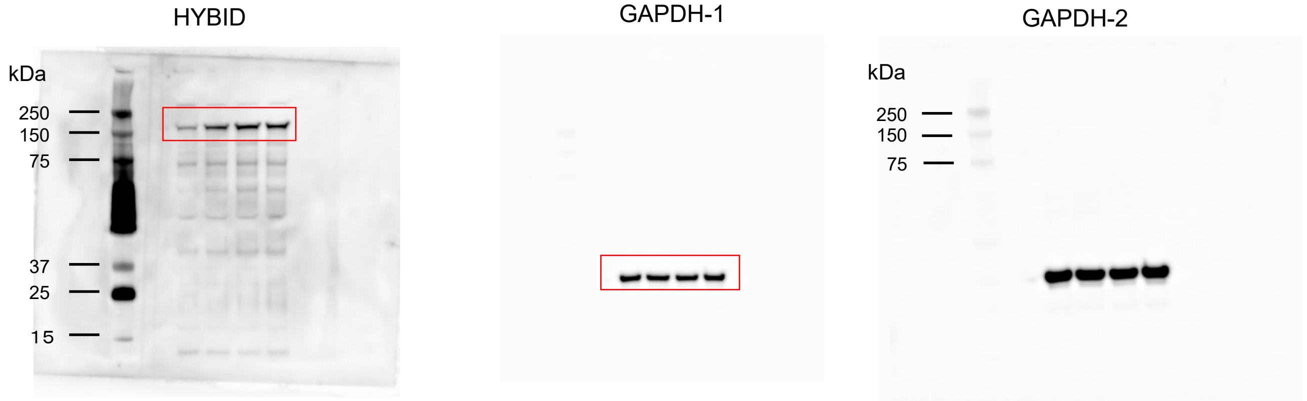

Full-length gels for **Figure 2c** (HYBID and GAPDH)

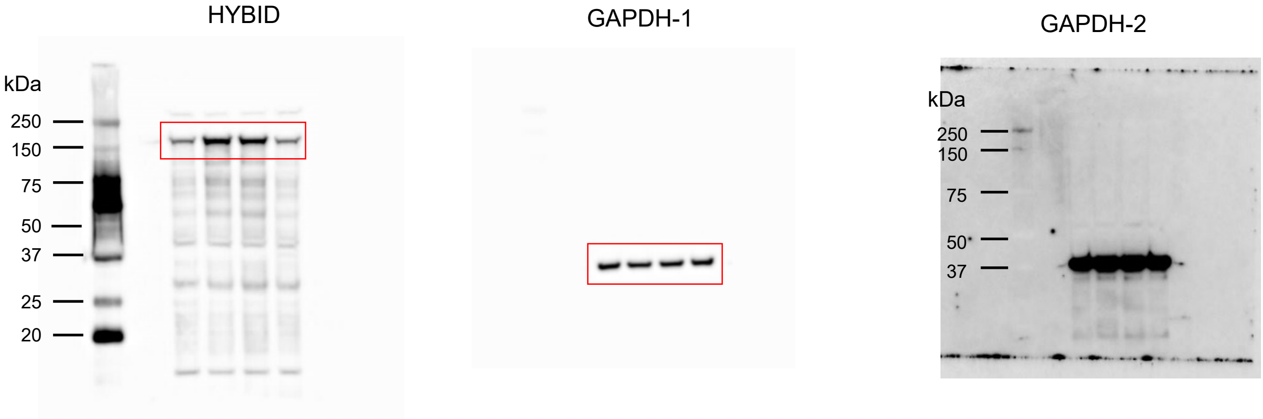

Full-length gels for **Figure 2e** (TMEM2 and GAPDH)

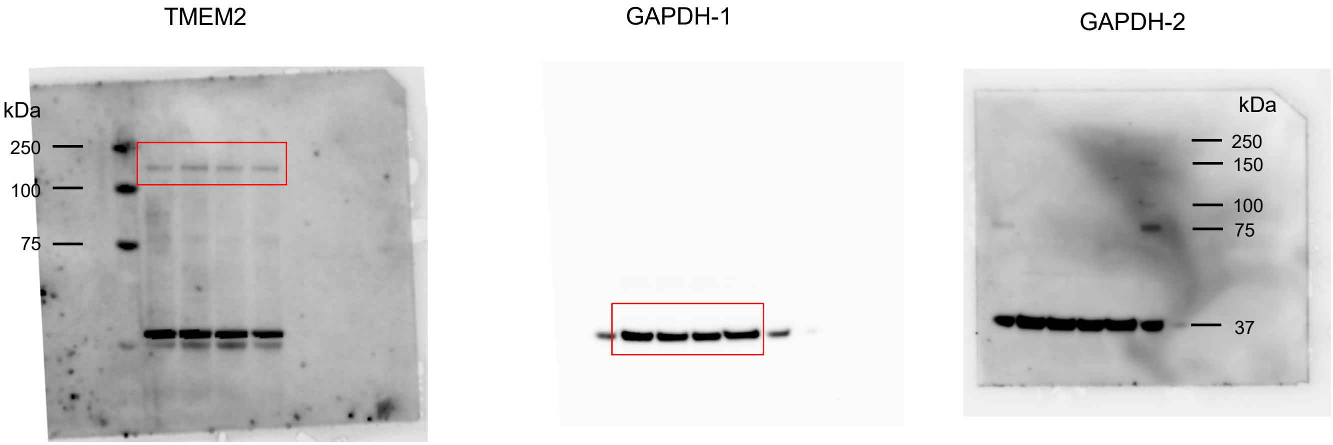

Supplementary Figure S3. Full-length gels corresponding to **Figure 2b, 2c and 2e**. For the final figures, Adobe Photoshop was used to crop the indicated bands (red lines) and the bands were placed in **Figure 2b, 2c** and **2e**. GAPDH-2 shows an image captured after a longer exposure. Immunoblotting for GAPDH of **Figure 2e** was performed by applying the same amounts of the samples to a different gel.

Supplementary Figure S4

Full-length gel for **Figure 3a** (HYBID and GAPDH)

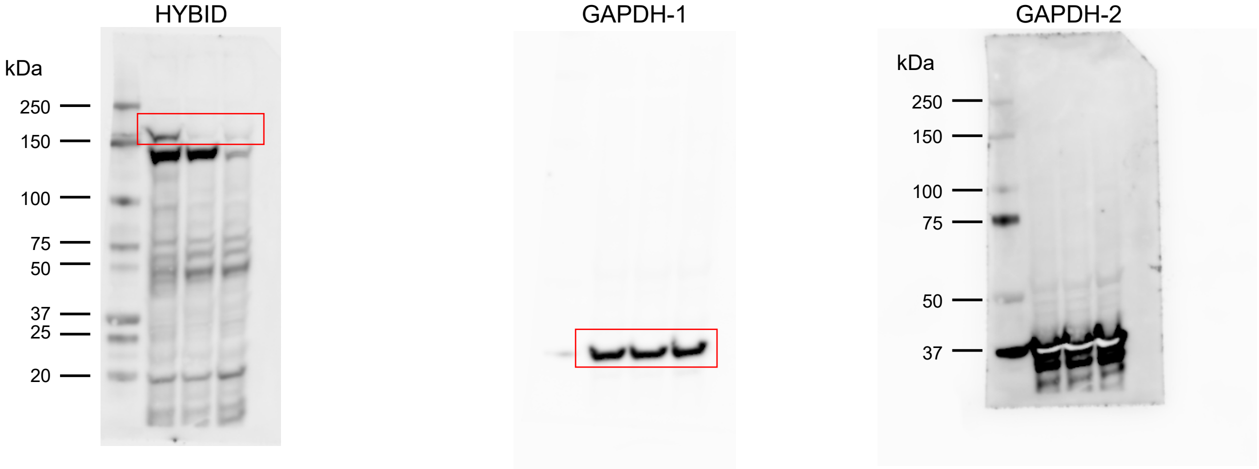

Full-length gel for **Figure 3b** (TMEM2 and GAPDH)

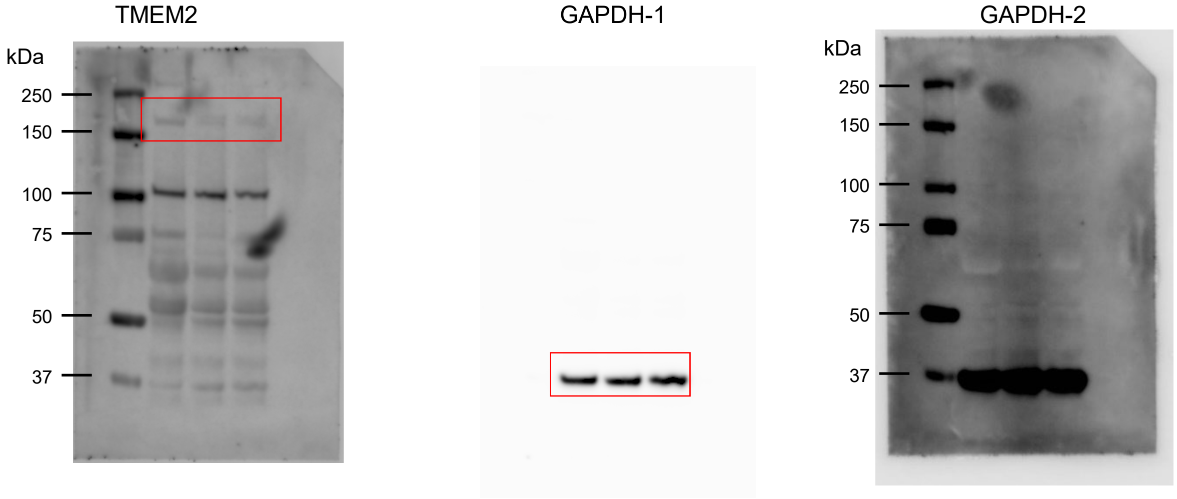

Full-length gel for **Figure 3c** (HYBID and GAPDH)

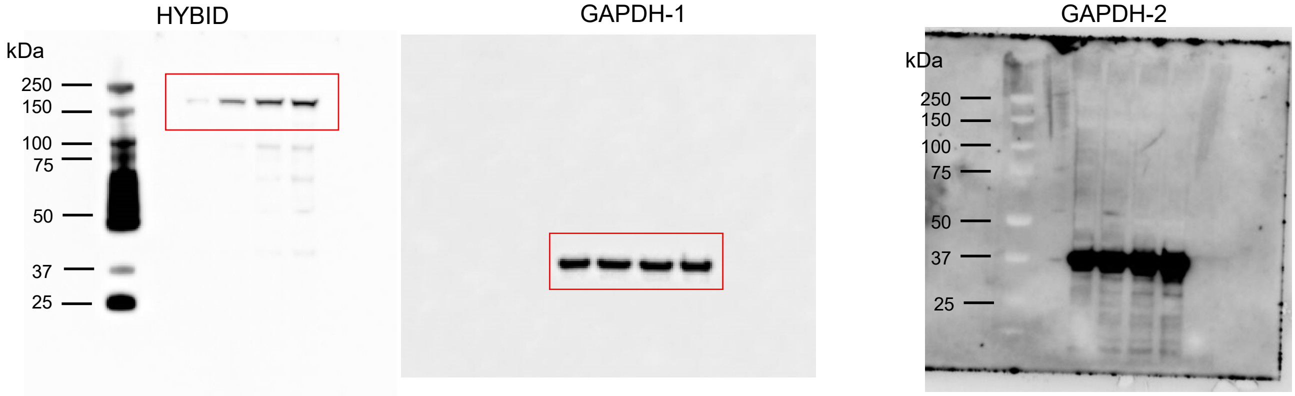

Supplementary Figure S4. Full-length gels corresponding to **Figure 3a, 3b and 3c**. For the final figures, Adobe Photoshop was used to crop the indicated bands (red lines) and the bands were placed in Figure **3a, 3b** and **3c**. GAPDH-2 shows an image captured after a longer exposure. Immunoblotting for GAPDH of **Figure 3a** was performed by applying the same amounts of the samples to a different gel.

Supplementary Figure S5

Full-length gels for **Figure 4b** (HYBID and GAPDH)

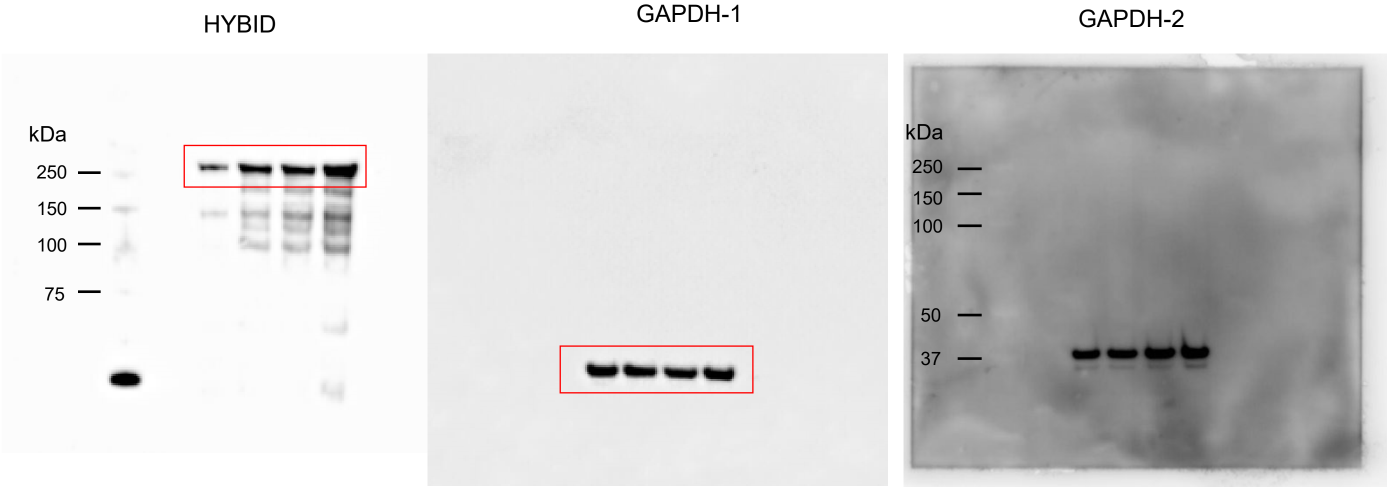

Supplementary Figure S5. Full-length gels corresponding to **Figure 4b**. For the final figures, Adobe Photoshop was used to crop the indicated bands (red lines) and the bands were placed in **Figure 4b**. GAPDH-2 shows an image captured after a longer exposure.

Supplementary Figure S6

Full-length gels for **Figure 5e, left** (HYBID and GAPDH)

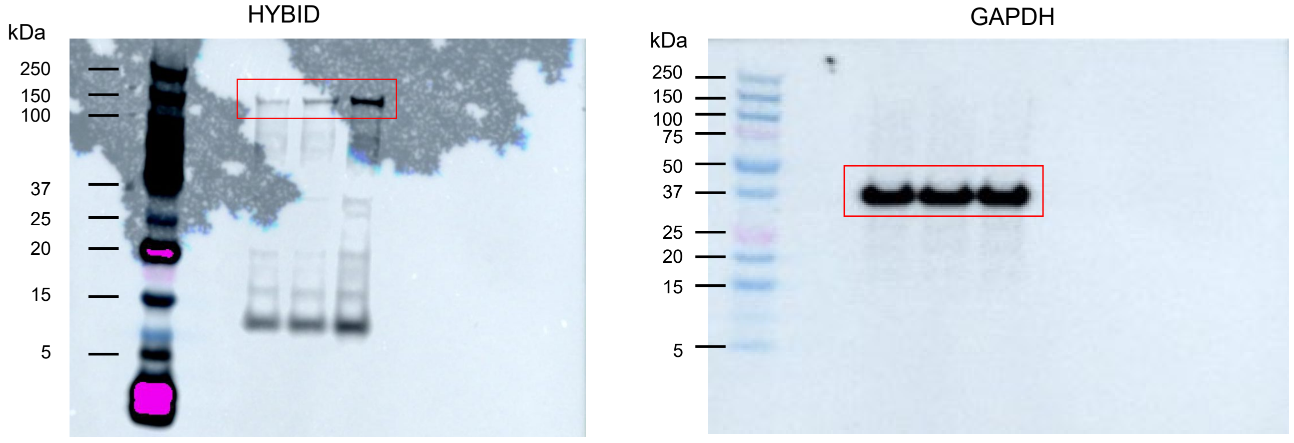

Full-length gels for **Figure 5e, right** (HYBID and GAPDH)

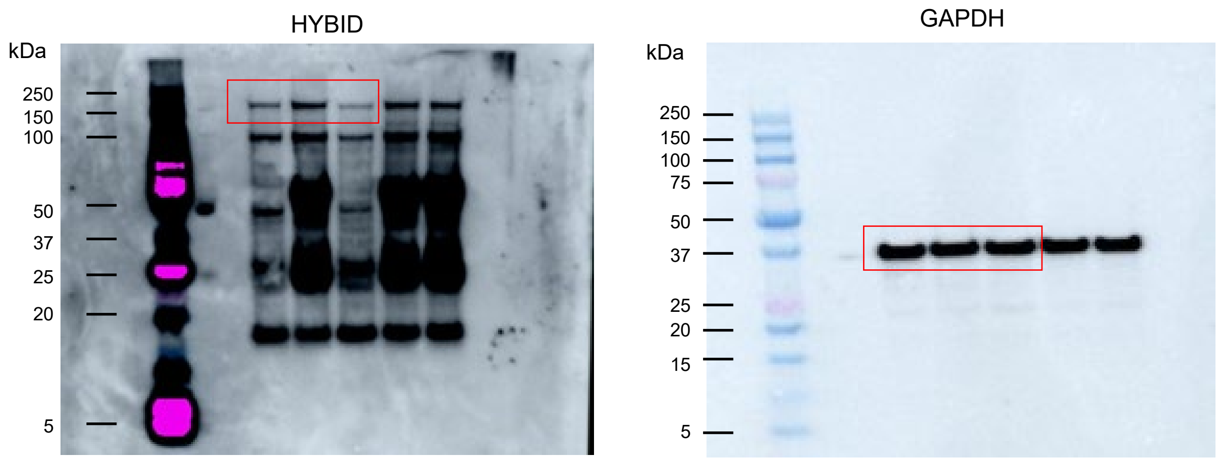

Supplementary Figure S6. Full-length gels corresponding to **Figure 5e**. For the final figures, Adobe Photoshop was used to crop the indicated bands (red lines) and the bands were placed in **Figure 5e**.
